# Top scoring pairs for feature selection in machine learning and applications to cancer outcome prediction

**DOI:** 10.1186/1471-2105-12-375

**Published:** 2011-09-23

**Authors:** Ping Shi, Surajit Ray, Qifu Zhu, Mark A Kon

**Affiliations:** 1 Harvard Medical School and Harvard Pilgrim Healthcare Institute, 133 Brookline Ave. Boston, MA 02215, USA; 2 Department of Mathematics and Statistics and Bioinformatics Program, Boston University, 111 Cummington St., Boston, MA 02215, USA; 3 Trilion Quality Systems, 500 Davis Drive, Suite 200, Plymouth meeting, PA 19462, USA

## Abstract

**Background:**

The widely used *k *top scoring pair (*k*-TSP) algorithm is a simple yet powerful parameter-free classifier. It owes its success in many cancer microarray datasets to an effective feature selection algorithm that is based on relative expression ordering of gene pairs. However, its general robustness does not extend to some difficult datasets, such as those involving cancer outcome prediction, which may be due to the relatively simple voting scheme used by the classifier. We believe that the performance can be enhanced by separating its effective feature selection component and combining it with a powerful classifier such as the support vector machine (SVM). More generally the top scoring pairs generated by the *k*-TSP ranking algorithm can be used as a dimensionally reduced subspace for other machine learning classifiers.

**Results:**

We developed an approach integrating the *k*-TSP ranking algorithm (TSP) with other machine learning methods, allowing combination of the computationally efficient, multivariate feature ranking of *k*-TSP with multivariate classifiers such as SVM. We evaluated this hybrid scheme (*k*-TSP+SVM) in a range of simulated datasets with known data structures. As compared with other feature selection methods, such as a univariate method similar to Fisher's discriminant criterion (Fisher), or a recursive feature elimination embedded in SVM (RFE), TSP is increasingly more effective than the other two methods as the informative genes become progressively more correlated, which is demonstrated both in terms of the classification performance and the ability to recover true informative genes. We also applied this hybrid scheme to four cancer prognosis datasets, in which *k*-TSP+SVM outperforms *k*-TSP classifier in all datasets, and achieves either comparable or superior performance to that using SVM alone. In concurrence with what is observed in simulation, TSP appears to be a better feature selector than Fisher and RFE in some of the cancer datasets

**Conclusions:**

The *k*-TSP ranking algorithm can be used as a computationally efficient, multivariate filter method for feature selection in machine learning. SVM in combination with *k*-TSP ranking algorithm outperforms *k*-TSP and SVM alone in simulated datasets and in some cancer prognosis datasets. Simulation studies suggest that as a feature selector, it is better tuned to certain data characteristics, i.e. correlations among informative genes, which is potentially interesting as an alternative feature ranking method in pathway analysis.

## Background

Gene expression profiling has been proved to be a valuable resource for classification of complex diseases such as cancer. Many studies have showed it is possible to extract compelling information from microarray data to support clinical decisions on cancer diagnosis, prognosis and response to treatment [1-6]. However, like other high-throughput studies, microarray data pose great challenges to accurate prediction in two respects. On one hand, there is a large amount of inherent noise and variability in samples, due to biological variations and experimental conditions, both of which can degrade prediction performance. On the other hand, difficulties also arise from high dimensionality (on the order of tens of thousands), as compared to a relatively small sample size (usually on the order of tens), which leads to the risk of over-fitting in many machine learning methods. This occurs especially in cases when the few training samples are not good representatives of classes, so that the classifier may learn inherent noise from irrelevant features in training data, leading to poor generalizability. Thus algorithmically speaking, there is a need for feature selection in order to improve model performance and avoid over-fitting.

Generally, feature selection methods in the context of gene expression studies can be divided into three categories: filter methods, wrapper methods, and embedded methods [7]. Filter approaches [8,9] involve calculating feature relevance scores, and selecting a subset of high-scoring features as input to the classifiers after removing low-scoring ones. They are computationally efficient and thus widely used. These approaches are also independent of classifier algorithms. On the contrary, wrapper and embedded approaches interact with classifiers by either wrapping around the classifiers [10-12], or being built within the classifier construction [13-15]. As a result, those approaches are usually much more computationally intensive than filters, and sometimes become so costly as to be impractical without pre-reduction of the search space with a filter method. Whereas filter techniques are frequently univariate in nature, assuming the features are independent and ignoring feature dependencies, wrapper and embedded techniques select features in a multivariate fashion by taking feature correlations into account, an approach which is certainly biologically relevant if we consider, e.g., how genes are co-regulated in pathways.

As more feature selection techniques are explored, computational efficiency and the ability to capture feature interactions remain important considerations. In 2004, Geman et al introduced the top-scoring pair (TSP) classifier [16], which was then refined and extended to *k*-TSP by Tan, et al [17], using k pairs of top ranking genes to build the classifier. There are several things worth noting in the feature ranking algorithm they employ. First, they replace actual expression levels with their ranks in each sample, which reduces inherent noise and is invariant to normalization procedures across platforms. Second, the idea of "relative expression reversals", on which the pairs are scored, captures a key mechanism of the disease process, which involves large differential changes of expression levels in up-regulated and down-regulated genes. Third, this scoring algorithm is multivariate at different levels, in the sense that bivariate interactions between features are exploited when genes are evaluated in pairs, and higher order interactions are addressed when the final ranking is derived from comparison of scores of all possible pairs. Last, by choosing reversed expression pairs, it is guaranteed that features from more than one cluster are collected, and the top-ranked features coming from different clusters can contribute orthogonal information to the classifier. With all the above unique qualities in its feature selection, *k*-TSP turns out to be a powerful classification method using simple comparisons, and in some cases, it is able to extract a single pair of genes for accurate diagnostic prediction. Nonetheless, given all its success in cancer diagnostic prediction, it has been noted that the performance of *k*-TSP is not always robust for some more difficult types, such as those involving cancer outcome prediction. This could be due to the complexity of data on one hand, and the relatively simple voting scheme of *k*-TSP classifier after the feature selection step on the other hand.

Compared to other complex machine learning methods, the support vector machine (SVM) is relatively insensitive to high dimensionality [18], though its performance can still improve considerably after feature selection [19]. Guyon et al. proposed the recursive feature elimination algorithm (RFE) [13], using the SVM weight vector to rank component features and discarding those with small weights in a recursive manner. This strategy is a multivariate approach embedded in the construction of the SVM classifier, and like other embedded methods, is relatively computationally intensive. Recently, Yoon et al reported their comparison of feature selection methods in combination with SVM in a number of cancer diagnostic datasets [20], suggesting that TSP may be an effective filter method candidate for other classifiers, if computational cost is not a concern.

In this paper, we describe a hybrid approach that integrates the TSP scoring algorithm into other machine learning methods such as SVM and *k*-nearest neighbors (KNN). A particular focus is placed on assessing this approach in a controlled environment by using simulated datasets with known properties, including correlation structures of informative genes (signal genes), the variance distribution in all genes, signal strength and sparsity, and sample size in the training set. We also apply SVM+TSP to four cancer prognostic datasets, and show that it achieves superior performance to *k*-TSP, and either outperforms or compares to that using SVM alone.

In general, different methods have their respective strengths and weaknesses in dealing with different aspects of data complexity, and a hybrid approach combining one algorithm with another can be beneficial [21,22]. This is also demonstrated in our study, both in real and simulated data, where sophisticated classifiers are enhanced by the feature selection scheme carved out from another learning algorithm.

## Results

### Simulated datasets

#### Simulation process

To investigate how feature selection methods respond to different data structures, we generated two types of data, with variations in aspects such as the strength of differentially expressed genes (signal genes), the sparseness of signal genes, and covariance structure. The basic model is as follows: each sample contains 1000 genes, of which 100 are signal genes. The signal genes follow the multivariate normal distribution *N*(*μ*, Σ) for class 1, and *N*(-*μ*, Σ) for class 2, with *μ *being a vector of 10 distinct values ranging from -0.25 to 0.25 with an increment of 0.05 or 0.1, each value being the effect size of 10 differentially expressed genes (denoted as *μ*_3_). The rest of the genes (900 noise genes) follow independent *N*(0, 1) distributions for both classes. For each simulation experiment, 150 independent samples were generated (75 for each class), from which 100 were randomly selected as the training set, with the remaining 50 used as the test set.

Based on this general model, the first type of data (Data-I) has all of the signal genes in one block Σ. The signal genes are sampled under an independent model or a correlated model. For the correlated model, the block has a compound symmetry (CS) structure with fixed variances of 1 and a common correlation coefficient *ρ*, as shown below:

$$\sum =\left[\begin{array}{ccccc} 1 & \rho & \rho & \ldots & \rho \\ \rho & 1 & \rho & \ldots & \rho \\ \vdots & \vdots & \ddots & \ddots & \vdots \\ \rho & \rho & \ldots & \rho & 1 \end{array}\right]$$

In the second type of data (Data-II), the signal genes come in 10 equal blocks, each consisting of 10 genes with a distinct value of effect size, and a CS correlation structure Σ_*i *_= Σ, so each Σ_*i *_has an equicorrelated structure. The blocks can be correlated among themselves by introducing an inter-block correlation coefficient *ρ*', which is always smaller than the within-block correlation *ρ *to ensure the covariance matrix is positive definite.

To mimic various situations in real datasets, we generated different variants of Data-I: Data-1b, Data-Ic, and Data-Id. Data-Ib is constructed based on the randomized variance model, with the variances drawn from an inverse gamma distribution, instead of taking on a fixed value of 1. Data-Ic and Data-Id reduce the proportion of signal genes from 10% to 1%, with Data-Ic containing the same *μ*_3 _vector as that in Data-I, and Data-Id containing a *μ *vector with a larger effect size, which has half of the values in the vector equaling -0.25, and the other half 0.25 (denoted as *μ*_3*b*_).

#### Comparison of TSP with Fisher and RFE as feature selection methods

We first compared the performance of SVM on Data-I and Data-II, using TSP, Fisher and RFE as feature selection methods. In each experiment, we applied the TSP, Fisher and RFE feature ranking algorithms to rank the genes, built SVM models with each level of selected genes on the training data, and then tested the models on the test set. Experiments were repeated 50 times to generate averaged results. Two aspects of performance are evaluated in the test data: the classification error rate at each level of gene selection, and the percentage of true signal genes recovered among the 30 and 60 top-ranked genes.

**Figure 1:**
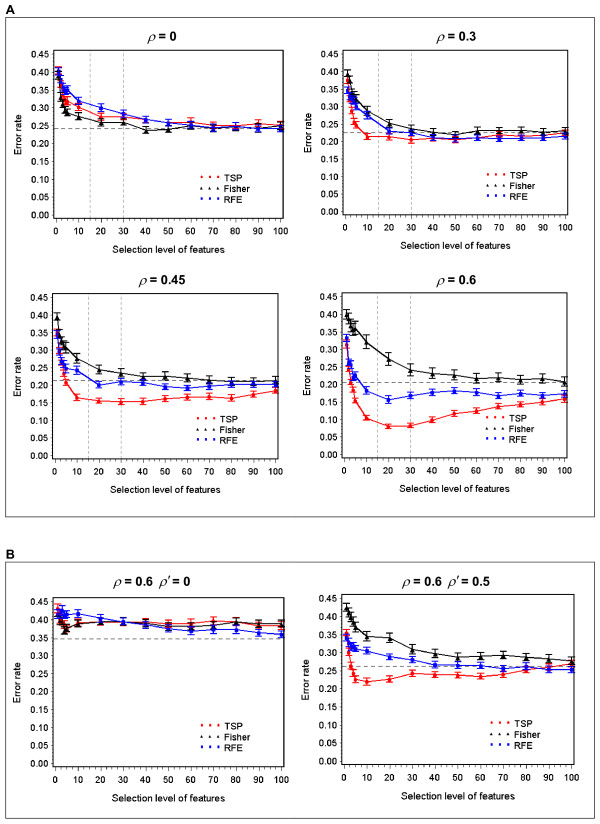
**Comparison of TSP, Fisher and RFE as feature selection methods for SVM as correlation varies among signal genes**. A) shows the error rates of SVM (mean ± SE) on the test set of Data-I (the single block structure) at various gene selection levels, as within-block correlation (*ρ*) varies. B) shows the error rates of SVM on the test set of Data-II (the multi-block structure), as inter-block correlation (*ρ'*) varies. The horizontal lines are the error rates of SVM using all features. The two vertical lines in A) show the number of pairs of genes in which recovery of signal genes are examined as shown in Figure 3.

Table 1 shows the classification error rates in Data-I at different levels of selected genes, with correlation coefficients at 0, 0.45 and 0.6. As can be seen, when the signal genes are independent, the different feature selection methods seem to perform comparably to one other (with Fisher slightly outperforming RFE), and the performance using all features is barely improved by using feature selection. However, as the signal genes become more correlated, TSP turns out to be increasingly advantageous over Fisher and RFE. We can take maximum error reduction (MER) as an indicator for the effectiveness of each method ((error rate at full feature level - minimum error rate at a selection level)/error rate at full feature level). When *ρ *= 0.45 the MERs are 28.5%, 1.9% and 10.3% for TSP, Fisher and RFE respectively, whereas at *ρ *= 0.6 they are 61.2%, -0.9%, and 24.3%, respectively. This trend is further illustrated in Figure 1A, which shows that in response to the progressively increased correlation among signal genes, the effectiveness of TSP gradually out-races Fisher and RFE, achieving increasingly lower error rates than those of the other two at most selection levels.

**Table 1:** SVM classification error rates on the test set of Data-I

	***ρ ***= **0**			***ρ ***= **0.45**			***ρ ***= **0.6**		
Level	TSP	Fisher	RFE	TSP	Fisher	RFE	TSP	Fisher	RFE
1	40.4	39.0	40.2	35.0	39.5	34.4	31.4	39.9	33.2
2	36.6	33.0	38.0	29.9	34.6	29.4	24.4	38.8	26.2
3	33.6	31.1	35.8	26.4	32.6	26.8	20.6	36.8	25.6
4	32.0	29.4	34.6	23.4	31.3	26.0	18.4	35.7	22.0
5	31.9	28.6	35.0	20.8	30.7	24.8	15.4	36.0	22.5
10	30.2	27.6	31.8	16.5	27.7	24.2	10.4	32.1	18.2
20	27.4	25.9	30.0	15.6	24.4	20.1	8.0	27.2	15.6
30	27.4	26.0	28.3	15.3	23.5	21.0	8.2	24.1	16.8
40	26.8	23.7	26.6	15.4	22.4	20.8	9.8	23.1	17.7
50	25.8	24.0	25.8	16.2	22.6	19.6	11.7	22.7	18.2
60	26.0	25.0	25.2	16.6	22.1	19.2	12.4	21.7	17.7
70	25.0	24.2	24.3	16.8	21.5	19.7	13.7	22.0	16.7
80	24.9	24.6	24.8	16.3	21.1	20.2	14.3	21.3	17.4
90	25.6	24.3	24.2	17.4	21.0	20.2	15.0	21.7	16.8
100	25.1	24.9	24.1	18.5	21.4	20.2	15.9	20.8	17.3
All features	24.1	24.1	24.1	21.4	21.4	21.4	20.6	20.6	20.6

The error rates (%, mean) are shown at various selection levels as correlation varies among signal genes, using TSP, Fisher and RFE as feature selection methods

When the signal genes are in the multi-block structure of Data-II, we do not observe the differential responses of the three feature selection methods as the signal genes within each block (within-block correlation *ρ*) become more correlated. However, if an inter-block correlation *ρ' *is introduced among all the blocks, we observe similar pattern of differentiated responses among the three methods. In Figure 1B, TSP, Fisher and RFE have comparable performance when the blocks are uncorrelated (*ρ *= 0.6, *ρ' *= 0). Nevertheless, when the blocks become correlated with one another, the effectiveness of these three methods diverges. The differentiation is more pronounced in the presence of a strong inter-block correlation (*ρ *= 0.6, *ρ' *= 0.5), where TSP is significantly better than Fisher and RFE at most selection levels.

**Figure 2:**
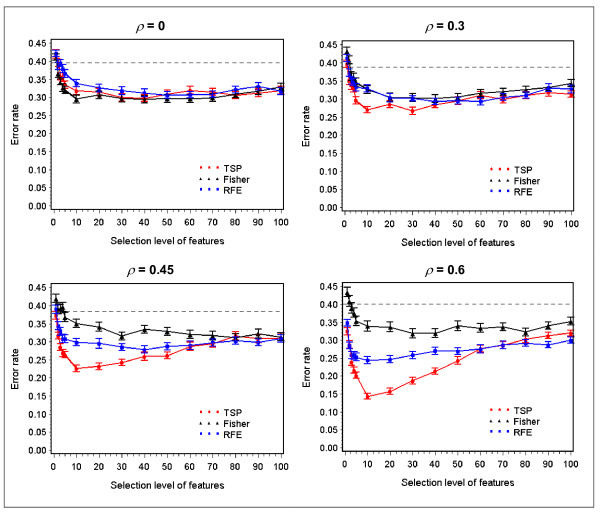
**Comparison of TSP, Fisher and RFE as feature selection methods for KNN as correlation varies among signal genes**. The error rates of KNN (mean ± SE) on the test set of Data-I, as within-block correlation (*ρ*) varies. The x-axis is the number of top ranked gene pairs for TSP, or half the number of top ranked genes for Fisher and RFE. The horizontal lines are the error rates of KNN using all features.

TSP, Fisher and RFE are also applied as feature selectors for another benchmark classifier *k*-nearest neighbors (KNN), with *k *= 3 as the number of nearest neighbors. As seen in Figure 2, again TSP, Fisher and RFE perform comparably when the signal genes in Data-I are independent, and all three improve on the performance of KNN using the entire set of features. As the correlation among signal genes become increasingly stronger, the performance of TSP starts to set apart from Fisher and RFE at *ρ *= 0.3, and is superior to the other two at most selection levels at *ρ *= 0.45, with the gap further widened at *ρ *= 0.6. In parallel, RFE also increases its performance in response to correlation, but is out-raced by TSP.

**Figure 3:**
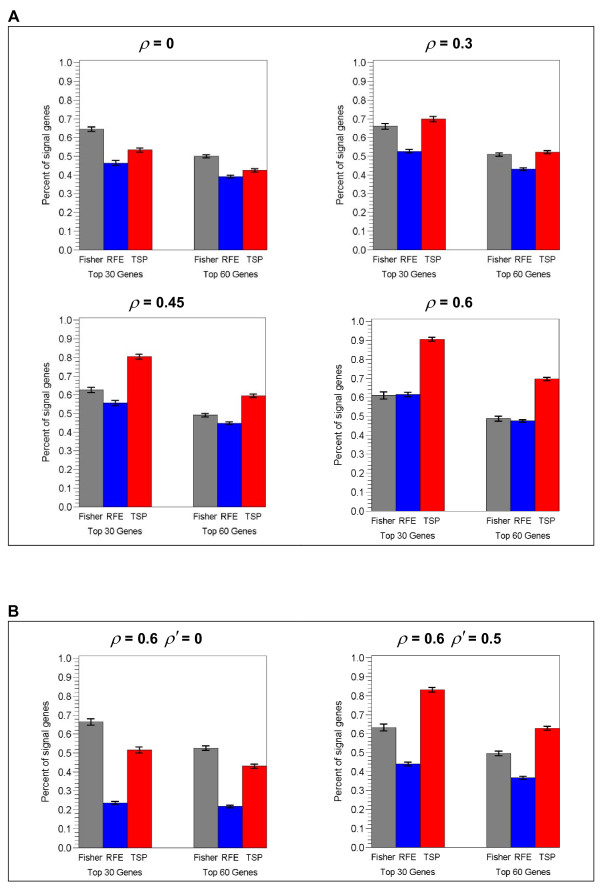
**Comparison of the recovery of signal genes by TSP, Fisher and RFE as correlation varies among signal genes**. The percentage (mean ± SE) of signal genes recovered in the 30 or 60 top-ranked genes by feature selectors TSP, Fisher and RFE, in A) as within-block correlation (*ρ*) varies in Data-I, and in B) as inter-block (*ρ'*) varies in Data-II.

In parallel with classification performance, the recovery of signal genes among the top-ranked genes by TSP, Fisher and RFE displays a similar trend in response to the increased correlation among the signal genes. We examine the percentage of signal genes in 30 and 60 top-ranked genes, based on the observation that most signal genes are recovered within top100 genes, and the recovery is well differentiated among the three methods in the top 30 and 60 genes. Figure 3 shows the percentage of the signal genes in the 30 and 60 top-ranked genes selected by the three ranking algorithms in Data-I and Data-II. In single block structure (Figure 3A), it can be seen that when the signal genes are uncorrelated, Fisher recovers slightly more signal genes than TSP and RFE (65% vs 53% and 46% respectively in the top 30 genes). As the correlation of signal genes progresses from 0.3 to 0.6, TSP gradually out-races Fisher to be the one that recovers most signal genes (from 53% to 91%), while the recovery rate for Fisher remains unchanged, and that of RFE increases to a smaller extent (from 45% to 61%). A similar trend is observed for multi-block data structures (Figure 3B), where the presence of inter-block correlation turns TSP into the leading feature selector for recovering signal genes.

#### Comparison of k-TSP+SVM with k-TSP and SVM

We compared the classification performance of TSP family classifiers, with our hybrid scheme using *k*-TSP as feature selection for SVM (*k*-TSP+SVM) in Data-I and Data-II, as well as some variants constructed based on Data-I (Table 2). In each experiment, the TSP ranking algorithm was used to rank the genes and build the model on training data at each level of selected genes through a standard leave one out cross-validation (LOOCV) procedure. The level that achieved the minimum LOOCV error rate was chosen as the size of gene subset, with which the classifier is built on the entire training set and then applied to the test data. Experiments were repeated 50 times to generate averaged test error rates, which were used to evaluate the performance of a classifier.

**Table 2:** Comparison of various classifiers in structural variants of Data-I and Data-II

A. Data-I of fixed variance vs. random variance with abundant signal genes										
**Data**	**Data structure**				**Classification error rate on the test set (%)**					

	**Signal genes**	**Variance**	**Correlation ρ**	**Signal vector**	**TSP**	***k*-TSP**	**SVM**	***k*-TSP + SVM**	**Fisher + SVM**	**RFE +** **SVM**

Data -I	10%	Fixed unit	0	*μ*_3_	39.2 ± 1.1	32.4 ± 0.9	**24.1 ± 1.0**	27.0 ± 1.1	26.5 ± 1.0	25.8 ± 1.1
Data -I	10%	Fixed unit	0.45	*μ*_3_	34.0 ± 1.0	21.7 ± 0.8	21.4 ± 0.9	**15.8 **± **0.9**	21.8 ± 1.0	21.0 ± 1.0
Data -I	10%	Fixed unit	0.6	*μ*_3_	31.0 ± 1.1	13.9 ± 1.0	20.6 ± 0.9	**10.0 ± 0.8**	21.9 ± 1.4	17.3 ± 1.1
Data -Ib	10%	Inverse gamma	0	*μ*_3_	26.1 ± 1.2	19.1 ± 1.1	26.6 ± 1.1	**12.1 ± 0.6**	12.4 ± 0.6	22.5 ± 0.8
Data -Ib	10%	Inverse gamma	0.45	*μ*_3_	18.0 ± 1.0	7.0 ± 0.5	23.7 ± 1.0	**3.4 ± 0.5**	5.4 ± 0.5	9.6 ± 1.0
Data -Ib	10%	Inverse gamma	0.6	*μ*_3_	15.8 ± 0.9	5.3 ± 0.5	23.8 ± 1.0	**1.6 ± 0.4**	4.2 ± 0.6	5.4 ± 0.7

**B. Data-I of stronger signal vs. weak signal with sparse signal genes**										

**Data**	**Data structure**				**Classification error rate on the test set (%)**					

	**Signal genes**	**Variance**	**Correlation ρ**	**Signal vector**	**TSP**	***k*-TSP**	**SVM**	***k*-TSP + SVM**	**Fisher + SVM**	**RFE +** **SVM**

Data -Ic	1%	Fixed unit	0	*μ*_3_	**46.5 ± 1.1**	49.4 ± 0.9	48.3 ± 1.0	47.8 ± 1.2	47.0 ± 1.1	46.8 ± 1.2
Data -Ic	1%	Fixed unit	0.45	*μ*_3_	44.1 ± 1.2	44.7 ± 0.9	45.8 ± 1.0	**43.1 ± 1.0**	45.6 ± 1.2	45.0 ± 1.2
Data -Ic	1%	Fixed unit	0.6	*μ*_3_	38.1 ± 1.5	43.2 ± 1.2	48.0 ± 1.1	**40.3 ± 1.2**	46.9 ± 1.2	41.7 ± 1.5
Data -Id	1%	Fixed unit	0	*μ*_3b_	43.5 ± 1.4	44.9 ± 1.1	43.7 ± 1.0	42.2 ± 1.3	**39.9 **± **1.1**	41.0 ± 1.0
Data -Id	1%	Fixed unit	0.45	*μ*_3b_	34.8 ± 1.2	36.8 ± 1.2	42.6 ± 0.9	**30.4 **± **1.3**	40.0 ± 1.2	35.0 ± 1.2
Data -Id	1%	Fixed unit	0.6	*μ*_3b_	30.4 ± 1.2	33.8 ± 1.4	40.8 ± 1.1	**23.0 **± **1.3**	38.1 ± 1.2	30.1 ± 1.3

**C. Data-II with independent blocks of signal genes vs. correlated blocks of signal genes**										

**Data**	**Data structure**				**Classification error rate on the test set (%)**					

	**Signal genes**	**Variance**	**Within-corr ρ**	**Inter-corr ρ'**	**TSP**	***k*-TSP**	**SVM**	***k*-TSP + SVM**	**Fisher + SVM**	**RFE +** **SVM**

Data-IIb	10%	Fixed unit	0.6	0	42.5 ± 1.1	34.7 ± 1.1	**34.6 **± **1.1**	37.9 ± 1.2	38.9 ± 1.0	37.6 ± 1.3
Data-IIb	10%	Fixed unit	0.6	0.5	33.4 ± 0.9	**22.9 ± 0.9**	26.2 ± 0.8	24.2 ± 0.9	30.6 ± 1.3	28.5 ± 0.9

The classification error rates (mean ± SE) of various classifiers as correlation varies among signal genes in A) Data-I of fixed variance vs. random variance when signal genes are abundant (10%); B) Data-I of strong signal vs. weaker signal when signal genes are sparse (1%); and C) Data-II of independent blocks vs. correlated blocks. The lowest error rates for each dataset are indicated in bolded.

Table 2A summarizes the classification performance of TSP, *k*-TSP, SVM, *k*-TSP+SVM, Fisher+SVM and RFE+SVM in Data-I and Data-Ib, the latter being a variant of Data-I whose variances follow an inverse gamma distribution with parameters a = 2 and b = 1. In Data-I, both *k*-TSP and *k*-TSP+SVM improve with increased correlation, with *k*-TSP+SVM (27.0%, 15.8%, and 10.0%) significantly outperforming *k*-TSP (32.4%, 21.7% and 13.9%) in all conditions. In contrast, SVM alone does not seem to pick up its performance as the correlation increases, and is thus increasingly outperformed by *k*-TSP+SVM when the correlation becomes stronger (21.4% and 20.6% vs 15.8% and 10.0%). Data-Ib, the dataset with a random variance structure, displays a similar trend, except that both *k*-TSP and *k*-TSP+SVM outperform SVM alone to a greater extent. It is noticeable that between the two TSP family classifiers, *k*-TSP is invariably superior to TSP. Meanwhile, RFE+SVM also improves with increased correlation in all cases, though much less robustly than *k*-TSP+SVM, whereas Fisher+SVM remains mostly unchanged in Data-I.

To investigate the impact of sparsity of the signal genes on classification, we created Data-Ic and Data-Id, which only contain one tenth as many signal genes as in Data-I. Interestingly, it is shown in Table 2B that as the percentage of signal genes is reduced from 10% to 1% in Data-Ic, the datasets become difficult for all the classifiers and none appears to be effective regardless of the presence of correlation. However, when the signal strength of the signal genes is increased from *μ*_3 _to *μ*_3*b *_in Data-Id, *k*-TSP+SVM steps over the others again, showing more robustness in rapidly improving its performance with increased correlation, and outperforming *k*-TSP and SVM at *ρ *= 0.45 (30.4% vs 36.8% and 42.6%), and *ρ *= 0.6 (23.0% vs 33.8% and 40.8%).

When signal genes are organized in multiple block structures, in which signal genes are correlated within each block (*ρ *= 0.6 for Data-IIb), a disparate picture emerges (Table 2C). When the blocks are uncorrelated with one another (*ρ *= 0.6, *ρ' *= 0), the performance of all the classifiers degrade drastically, and *k*-TSP+SVM does not show any advantage. In contrast, when the blocks are correlated (*ρ *= 0.6, *ρ' *= 0.5), each classifier significantly improves its performance, with *k*-TSP, and *k*-TSP +SVM achieving comparable best performances (22.9%, and 24.2%).

#### The effect of sample size in training data

**Figure 4:**
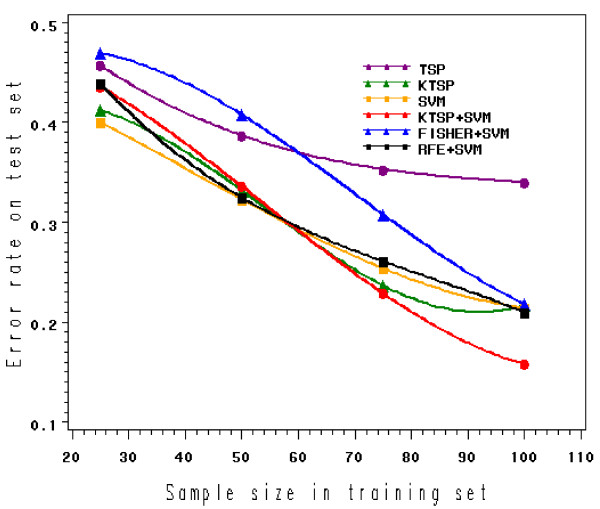
**Comparison of various classifiers in Data-I with different sample sizes in the training set**. The classification error rates (mean) on Data-I (*ρ *= 0.45), with the training sets containing different sample sizes (n = 25, 50, 75, 100).

In many microarray studies, sample sizes in training sets are usually limited. It has been suggested that the TSP ranking algorithm is sensitive to the perturbation of training samples [17]. To assess this effect by simulation, we generated datasets of Data-I with sample sizes of 25, 50, 75 and 100 in the training sets, with signal genes moderately correlated (*ρ *= 0.45). Experiments were repeated 50 times to generate averaged results. As shown in Figure 4, all the classifiers improve their performance as the sample size increases. When the training size is only 25, the performances of all classifiers deteriorate, indicating it almost becomes impossible to train a classifier with such a small training size. As the training set becomes larger, TSP and Fisher+SVM appear to be significantly less effective than the rest, in which *k*-TSP+SVM is relatively comparable to others. As the sample size reaches 100, *k*-TSP+SVM (15.8%) rises above all others, significantly outperforming TSP (34.0%), *k*-TSP (21.7%), SVM (21.4%), Fisher+SVM (21.8%), and RFE+SVM (21.0%) (Figure 4 and additional file 1).

### Real datasets

#### Cancer prognostic datasets

We applied the above hybrid scheme *k*-TSP+SVM to four cancer prognostic datasets, all of which are available on our project website, and the information of these datasets is summarized in Table 3. The first dataset is van't Veer's breast cancer dataset [3], obtained from Rosetta Inpharmatics, which is already partitioned into training and test data. The training data consists of 78 patients, 34 of whom developed distant metastases or died within 5 years (poor prognosis), with the rest consisting of those remained healthy for an interval of more than 5 years (good prognosis). The test data consists of 19 patients, 12 with poor prognosis and 7 with good prognosis. Since this dataset contains many missing values, certain pre-processing was performed. First, two samples (one from each prognosis) with more than 50% of missing gene values in the training data were removed. Next, any genes whose value was missing in at least one sample was discarded, amounting to a total of 3.5% of all genes. The log-transformed ratio of the two channels was used for analysis.

**Table 3:** Information of cancer prognosis datasets

Dataset	Number of genes	Number of samples (training/test)	Poor prognosis	Good prognosis	References
van't Veer Breast cancer	23624	76/19	33/12	43/7	van't Veer et al. (2002)
Wang Breast cancer	22283	209	71	138	Wang et al (2005)
Lung adenocarcinoma	7129	86	24	62	Beer et al. (2002)
Medulloblastoma	7129	60	21	39	Pomeroy et al. (2002)

Another breast cancer dataset is derived from Wang et al [6], and contains a subset of ER-positive, lymph-node-negative patients who had not received adjuvant treatment. We used the raw intensity Affymetrix CEL files and normalized the data by RMA procedures using Bioconductor packages http://www.bioconductor.org, obtaining a final expression matrix comprising 22283 features and 209 samples. Again patients who developed distant metastases or died within 5 years are classified as poor prognosis subjects, and those who remained healthy for more than 5 years as good prognosis ones. The dataset consists of 71 patients with poor prognosis, and 138 with good prognosis

The other two cancer prognostic datasets are obtained from the cancer dataset depository of the Broad Institute. One is a dataset of 86 patients with primary lung adenocarcinoma, which consists of 62 patients who were alive, and 24 patients who had died [4]. The other is a dataset of 60 patients with medulloblastomas, which consists of 39 survivors and 21 treatment failures after radiation and chemotherapy [5]. Both datasets are pre-processed and contain 7129 genes.

#### Application to cancer prognostic datasets

The classification performance of *k*-TSP+SVM is compared with *k*-TSP and SVM in the three cancer prognostic datasets. We used the independent test set when it was available from the original dataset (van't Veer dataset); otherwise we performed 5-fold cross-validation and averaged the results from two 5-fold experiments.

Table 4 summarizes the results using different methods on the above prognostic datasets. It is noteworthy that *k*-TSP is invariably less robust on this type of data, consistent with previous observations (unpublished data), although it does seem that its performance improves as sample size increases. In the van't Veer breast cancer dataset, *k*-TSP+SVM significantly improves the performance, which only makes two errors on the 19-case test set, achieving an error rate of 10.5%, as compared to 47.3% with *k*-TSP and 31.6% with SVM alone. In the other datasets, nonetheless, the extent of improvement of *k*-TSP+SVM over *k*-TSP appears to be related to sample size. In cases where sample size is small or moderate (adenocarcinoma and medulloblastoma), *k*-TSP+SVM improves considerably over *k*-TSP (27.8% versus 40.7%, 35.7% versus 49.6%, respectively); while in the case where sample size is large (Wang dataset), the improvement is moderate (32.9% versus 37.3%). In comparison to SVM, on the other hand, *k*-TSP+SVM achieves similar performances in all three cases, while using a small number of genes as opposed to the entire set of genes. In the two 5-fold experiments, *k*-TSP+SVM utilizes an average of 61 pairs of genes in Wang breast cancer dataset, 57 pairs in the lung adenocarcinoma dataset, and 76 pairs in the medulloblastoma dataset.

**Table 4:** Comparison of various classifiers in cancer prognosis datasets

Dataset	Error rate on 2X 5-fold CV (%)				Error rate on the test set (%)			
	**TSP**	***k-*TSP**	**SVM**	***k*-TSP+SVM**	**TSP**	***k*-TSP**	**SVM**	***k*-TSP+SVM**

van't Veer Breast cancer					68.4	47.3	31.6	10.5
Wang Breast cancer	41.4 ± 2.5	37.3 ± 2.8	30.1 ± 1.8	32.9 ± 3.0				
Lung adenocarcinoma	41.2 ± 2.5	40.7 ± 2.5	29.7 ± 3.5	27.8 ± 2.9				
Medulloblastoma	48.3 ± 4.3	49.6 ± 7.4	37.5 ± 5.9	35.8 ± 4.8				

In the van't Veer breast cancer dataset where there is an independent test set, the error rate on the test set was obtained at the gene selection level at which the training set achieves its minimum LOOCV error rate. In the other datasets where there is no separate test set, the error rates (mean ± SE) were obtained from two experiments of five-fold cross validation.

**Figure 5:**
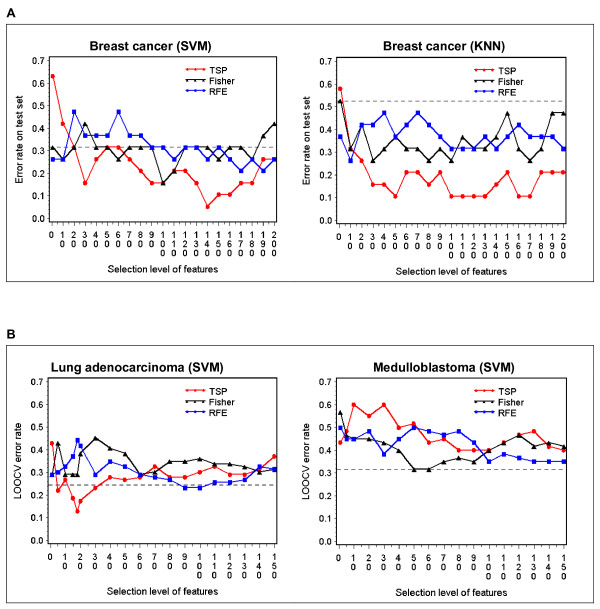
**Comparison of TSP, Fisher and RFE as feature selection methods in the cancer prognostic datasets**. A) shows the SVM and KNN classification error rates on the test set of van't Veer Breast cancer dataset at various gene selection levels, using TSP, Fisher and RFE as feature selection methods. B) shows LOOCV error rates by SVM in Lung adenocarcinoma and Medulloblastoma datasets at various gene selection levels, using TSP, Fisher and RFE as feature selection methods. The x-axis is the number of top ranked gene pairs for TSP, or half the number of top ranked genes for Fisher and RFE. The horizontal lines are the error rates of SVM or KNN using all features.

Meanwhile, we compared TSP for feature selection with Fisher and RFE, using both SVM and KNN as classifiers in some datasets. For the breast cancer dataset where a separate test set is available, the error rate was obtained directly on the test set at selected levels (pairs) of genes. For the lung adenocarcinoma and medulablastoma datasets, on the other hand, standard LOOCV error rate was estimated at selected level of genes. Interestingly, different patterns of response are observed in different datasets when feature selectors are combined with a classifier. In the breast cancer dataset (Figure 5A), TSP has significantly lower error rates than Fisher and RFE at most levels of selected genes, using either SVM or KNN as the classifier. As compared to 31.6% achieved by SVM without gene selection, the lowest error rate of 5.3% is achieved for *k*-TSP+SVM on the top 140 pairs. In parallel, as compared to 52.6% achieved by KNN without gene selection, an error rate of 10.5% is achieved for *k*-TSP+KNN using the top 50 pairs. On the other hand, in the lung adenocarcinoma dataset (Figure 5B), the performance of TSP as a feature selector is set apart from that of Fisher and RFE mainly within the top 60 pairs. *k*-TSP+SVM achieves its minimum error rate of 12.8% using the top 18 pairs, which is a sizable improvement upon the 24.4% error rate by SVM without gene selection. Finally, the medulablastoma dataset presents yet another scenario (Figure 5B). None of the feature selection methods appears to be effective, and no improvement is observed at any level of selected genes as compared to the performance by SVM without gene selection.

## Discussion

Results from simulated and real datasets show that the *k*-TSP feature ranking algorithm can be integrated usefully into machine learning classifiers for feature selection. The hybrid algorithm outperforms *k*-TSP, and in some cases the corresponding classifier using all features in cancer prognostic datasets. Simulation studies suggests that with certain data characteristics, this ranking algorithm appears to be a superior feature selector to the univariate Fisher method, as well as the multivariate recursive RFE procedures embedded in SVM.

In assessing the effectiveness of a new method, simulated and real datasets play complementary roles. Simulated datasets with known properties can be used for exploring the robustness and parameter space of a given method, and for studying the influence of data characteristics on its performance. It can also provide insights regarding its strengths and weaknesses in real situations. Yet, due to the largely unknowable properties of gene expression data, e.g. the true distribution of expression values across genes in different biological states or the exact correlation structures within and among all the gene networks, simulation is usually an over-simplified representation of real scenarios, and unavoidable biases can be introduced by specified distributions and model assumptions. For that reason, the effectiveness of a method suggested by simulation needs to be validated on real data. On the other hand, the sample size limitation in real datasets may impede the detection of the true data structure, and thereby the demonstration of the advantage of a method in tune with that structure, whereas simulation allows generation of a large number of samples for the full manifestation of data characteristics.

By constructing simulated datasets with various structures, we were able to observe how different feature selection methods interact with different data properties, including the correlation structure in signal genes, signal strength and sparsity, as well as sample size in the training set. Indeed, our simulated data sets *D *fall into a parameter space *A *whose dimensions consist of (1) the total sample size *n*, (2) the proportion *q *of signal genes, (3) the signal strength *s *of such genes, (4) the number *N *of blocks, (5) inter-gene correlation *ρ *within blocks, and (6) inter-block correlation *ρ'*. Within the space *A *this study can be viewed as a Monte-Carlo procedure determining which data sets *D *within *A *are best tuned to a feature selection method in combination with a classifier. In theory, extension of this procedure to a fuller exploration of the space *A *may lead to the possibility of taking a biological dataset *D*' and determining (from within the training set) a point *D *in *A *which *D*' falls closest to, if the above parameters from *D*' can be empirically measured, and thus estimating which combination of feature selection and classifier is the best match to *D*'. However, there remain many challenges in mapping a real dataset in the parameter space, one of them being the attempt to extract the true correlation structures within and among all gene networks, especially in cases of small sample sizes.

Among the correlation structures considered here, the simplest version is the single-block design with all signal genes in one covariance matrix with uniform inter-gene correlation. In this case we found that TSP, Fisher and RFE perform comparably when the signal genes are independent (*ρ *= 0). However, as the signal genes become increasingly correlated, TSP appears to improve increasingly over Fisher and RFE, both in terms of classification accuracy and the recovery of signal genes (Figure 1A, Figure 3A). It is notable that the univariate Fisher method seems to be steady regardless of correlation, so that its performance becomes inferior to the other two as the correlation progresses. This indicates that correlated data are more in tune with multivariate methods such as TSP and RFE, which select features based on the joint information from multiple signal genes, rather than the differential expression of individual signal genes. Interestingly, between the two multivariate approaches, it is the simple TSP algorithm, which is less computationally costly, that responds to the correlation more robustly and achieves a better performance. A similar trend was also observed in a more complex version involving multi-block design, with signal genes divided into 10 covariance blocks. As these blocks become increasingly correlated with one another, TSP seems to become an increasingly superior feature selector to Fisher and RFE (Figure 1B). It is worth mentioning that in an extensive study comparing univariate and multivariate feature selection methods on seven biological datasets, Lai et al. found that most of the multivariate ones do not result in improvement over the univariate ones [23]. The above simulation result in fact does reveal an advantage of a multivariate approach in the presence of data correlations, and it is worth investigating why this has not been observed correspondingly in real datasets, where many differentially expressed genes are co-regulated in pathways. One possible reason could be, as the authors stated, the limited sample sizes in real datasets, which makes a correlation structure difficult to extract.

Therefore, correlation among signal genes influences the performance of various classifiers using TSP algorithm for feature selection. As shown in Table 2A, k-TSP+SVM outperforms *k*-TSP when the signal genes are independent. As the correlation among signal genes rises, the performances of both classifiers are increasingly improved, mainly due to the increasing effectiveness of their shared ranking algorithm. Thus the difference between *k*-TSP+SVM and *k*-TSP tends to taper off as signal genes become more correlated. SVM alone, on the other hand, appears to remain constant in response to the increased correlation. As a result, the difference between *k*-TSP+SVM and SVM is enlarged as signal genes become more correlated.

An interesting notion was introduced by Jin et al. [24], showing that in the parameter space of real data, there exists a region in which successful classification is virtually impossible, this region being jointly determined by the fraction *q *of discriminating features (signal genes), the strength *s *of those features, and the ratio of sample size *n *to feature number *p*. This is significant because it establishes a limit on separability for datasets that are difficult to classify. In our simulation experiments, we manipulated the parameter space by varying the fraction and strength of signal genes, the training set sample size, and correlation among signal genes, so as to observe how classifiers respond to the changes in the parameter space. For example, in Data-I, where the signal strength is relatively high and signal genes relatively abundant (10%), *k*-TSP+SVM seems to perform significantly better than *k*-TSP, SVM, and other classifiers in most cases, especially when signal genes are correlated (Table 2A). However, when signal genes become very sparse (1%) in Data-Ic, *k*-TSP+ SVM loses its advantage over the other classifiers in either uncorrelated or correlated data, and its performance deteriorates severely like all of the others. Nevertheless, when the sparse signals increase their signal strength in Data-Id, *k*-TSP+SVM regain it robustness and superiority to other classifiers in correlated data (Table 2B). Finally, the sample size in the training sets proves to be crucial. In Data-I with signal genes moderately correlated, *k*-TSP+SVM significantly outperforms all the other classifiers when sample size is relatively large (n = 100), but only slightly outperforms *k*-TSP and SVM when the sample size becomes smaller (n = 75), and totally losses its advantage when sample size is very small (n = 25), at which point the performances of all classifiers deteriorate (Figure 4).

The above results suggest that as datasets fall closer to the region of inseparability, in which features are rare and weak, or the sample size is small, *k*-TSP+SVM losses its superiority with respect to other classifiers, and in fact no classifier built from the data themselves is likely to separate the two classes well.

In actual cancer microarray datasets, the data characteristics, and as a result the difficulty of classification, largely depend on the types of data. In general, diagnostic datasets usually contain a set of salient pathophysiological entities that can be easily used to distinguish between cancer and normal tissues with a number of algorithms. Prognostic datasets, on the other hand, are more challenging, since the samples with poor and good prognoses often share the same pathophysiological characteristics, and the features that differentiate between the two classes are relatively sparse and not well defined. Our observations as well as those of others show that compared to its robustness in cancer diagnostic datasets, *k*-TSP seems to be less successful in datasets involving cancer outcome prediction. This may partly be due to the relatively simple voting scheme that does the decision-making of the classifier, given that the feature selection algorithm is very effective. Thus we believe that in such cases performance can be improved with a hybrid scheme, in which the *k*-TSP ranking algorithm is combined with a powerful and multivariate machine learning classifier such as SVM. This is confirmed in the breast cancer dataset (Table 4), where the test error is reduced from 47.3% with *k*-TSP to 10.5% with *k*-TSP+SVM. Notably SVM benefits from the feature reduction as well, since with the entire set of features its error rate is 31.6%. The performance of *k*-TSP is also significantly improved with *k*-TSP+SVM in the lung adenocarcinoma and medullablastoma datasets (Table 4), although in both cases SVM alone achieves comparable performances. On the other hand, consistent with what is observed in simulated data with correlated signal genes, TSP is a superior feature selector to Fisher and RFE in both the breast cancer and lung adenocarcinoma datasets (Figure 5).

## Conclusion

An effective feature selection method is crucial in classification and prediction of complex diseases through gene expression analysis. We integrated the feature ranking algorithm of *k*-TSP with multivariate machine learning classifiers, and evaluated this hybrid scheme in both simulated and real cancer prognostic datasets.

We compared the TSP ranking algorithm with a univariate feature selection method Fisher, and a multivariate method RFE in simulated data. In the model where the signal genes are uncorrelated, the three feature selectors perform comparably in terms of classification accuracy, with Fisher recovering more signal genes. In the models where signal genes are increasingly correlated, however, TSP increasingly outperforms Fisher and RFE, both in terms of the classification accuracy and recovery of signal genes. We also observed that as classifiers, *k*-TSP+SVM outperforms *k*-TSP in most cases, and significantly improves the performance of SVM alone when signal genes are correlated.

This hybrid scheme was applied to four cancer prognostic datasets. *k*-TSP+SVM outperforms *k*-TSP in all datasets, and achieves either comparable or superior performance to that using SVM with all features. As observed in simulated data, TSP appears to be a superior feature selector to Fisher and RFE in two datasets.

We conclude that the TSP ranking algorithm can be used as a computationally efficient, multivariate filter method for feature selection in machine learning. Simulation studies suggest that this algorithm is better tuned to correlated signal genes, the implication of which should be further explored in real datasets, where differentially expressed genes act in concert due to pathway dependencies. Moreover, as Sexena et al. showed [25], many pathways include both up- and down-regulated components. As a ranking algorithm that is very effective in capturing up-regulated and down-regulated genes simultaneously, TSP ranking can possibly be used as an alternative method to generate the rank list in gene set enrichment analysis, which may reveal a unique profile of enriched sets of genes.

Our preliminary work in single enrichment analysis suggests that, among the subsets of top-ranked genes selected by the three feature selectors from lung adenocarcinoma dataset, those by TSP are most relevant to cancer related pathways. We plan to explore this further to see if TSP ranking algorithm has a distinct advantage in revealing important signatures genes in some real datasets.

## Methods

### Feature Selection Methods

#### Fisher criterion (Fisher)

Univariate approaches of feature selection evaluate how well an individual feature discriminates between two classes according to a criterion such as the *t*-statistic, weighted-voting [26], or a measure similar to Fisher's discriminant criterion [27]. For univariate ranking we adopt the Fisher criterion, based on the correlation score defined as

$$\frac{{\left(\mu_{i}\left(+\right)-\mu_{i}\left(-\right)\right)}^{2}}{\sigma_{i}{\left(+\right)}^{2}+\sigma_{i}{\left(-\right)}^{2}}$$

with the numerator representing the square of difference in means of two classes(+ and -), and the denominator representing the sum of the square of their variances. The genes are ranked according to their Fisher scores, from the most to the least informative. This ranking determines the ordering of gene subsets to be evaluated, e.g. the first five genes, the first 10 genes, etc. In combination with a machine learning classifier, the informativeness of each gene set is evaluated by a leave-one-out cross-validation (LOOCV) procedure in the training set, so that the most informative subset will be found based on the classification performance within the set. To validate the performance on a test set, the classifier is built with the selected gene sets from training and then applied to the test set.

We used an object-oriented machine learning package (Spider, [28]) to perform Fisher ranking in combination with machine learning classifiers.

#### Recursive feature elimination (RFE)

RFE is an iterative backward elimination procedure embedded into a SVM classifier [13]. The decision function of SVM for an input feature vector **x **= (*x*_1_, ..., *x*_*p*_) is:

(1) D(x)=sgn{w⋅x+b}

where $\left(w_{1},\ldots ,w_{p}\right)=w=\overset{n}{\underset{i=1}{\sum }}\alpha_{i}y_{i}x_{i}$, is the vector of feature weights, **x***_i _*is the feature vector and *y_i _*the class (± 1) of the of the *i^th ^*subject in the training set, and *α_i _*is obtained from a quadratic programming optimization algorithm with inputs {x_i_, *y_i_*} from the training set. The magnitude, ${w_{i}}^{2}$ of the *i^th ^*component is employed to rank the corresponding features in the input x. At each iteration, the classifier (1) is built on the training set, assigning a weight *w_i _*to gene *i*. Then one gene (or some fixed proportion of genes) with the smallest weight is removed, and the weights *w_i _*are re-calculated on the smaller set of remaining genes. The final gene ranking is constructed by placing initially eliminated genes at the bottom of the list, and subsequently adding genes eliminated later. Since the weight assigned to a gene depends on all other genes in a given iteration, a gene is not evaluated individually, but in relation to a group of genes. Therefore, RFE is a multivariate backward search, which is also a computationally intensive process as it involves training a new classifier at each subsequent subset of features.

We used the Spider package to perform RFE ranking in combination with SVM. The parameters are set in so that features are removed 10% at a time until there are 500 features left, after which a single feature is removed at each iteration.

#### Top-scoring pairs (TSP)

The scoring algorithm for the *k*-TSP classifier is based on relative gene expression ordering, as well as an exhaustive pairwise search [16]. Let *R_in _*denote the rank of the *i^th ^*gene in the *n^th ^*sample, and consider the rank matrix **R **= (*R_ij_*). Genes are evaluated in pairs, scored by their differences in the probabilities *P*(*R_i _*<*R_j_*) between class C1 and class C2, formally defined as the difference of the following conditional probabilities:

$$\Delta_{ij}=|P\left(R_{i}<R_{j}|C1\right)-P\left(R_{i}<R_{j}|C2\right)|$$

Then Δ*ij* is used as a criterion to produce a ranking of gene pairs, and a series of gene pairs is established, determining the order in which they are to be subsequently evaluated.

As mentioned, the TSP feature ranking is a multivariate approach, since genes are not evaluated individually, but in pairs. Meanwhile, the selection of each pair results from comparing it with all other possible pairs involving neither of the given pair of genes. An obvious advantage of this algorithm is that it is rank-based, so that it is invariant to pre-processing such as scaling and normalization. Notably, compared to some more complex multivariate searches, TSP ranking is relatively simple and hence more computationally efficient.

We adopted the scoring algorithm of the Matlab version of TSP and integrated it with the classification evaluation framework implemented in a Matlab environment.

### The machine learning and *k*-TSP classifiers

#### Support vector machine (SVM)

SVMs are powerful and elegant linear classifiers [18] which have been highly successful in many applications. Essentially, a SVM maps training data points ${\left\{x_{i}\right\}}_{i=1}^{n}$in an input space ℝ^p ^to vectors *ϕ*(**x**_i_) ∈ *F*, where *F *is a higher dimensional feature space. Such a mapping is compactly specified using a kernel matrix $K_{ij}=\left\langle \phi \left(x_{i}\right),\phi \left(x_{j}\right)\right\rangle$ whose entries are dot products of the images in F. The resulting linear structure in *F *allows construction of a discrimination surface *H*: **w **· **x **+b = 0 (which is a hyperplane in the variable *ϕ*(**x**) in *F*) best separating the images of the two classes in *F*, using a maximum margin criterion. Here **w **is a vector perpendicular to *H*, and *b *determines the displacement of *H*. This criterion determining **w **and *b *involves choosing *H *in a way which minimizes the weighted sum of misclassified points within a fixed margin (distance) *d *from *H*, done by solving a convex optimization problem. The result is a discriminant function *D*(*x*) = **w **· **x **+ **b**, whose sign determines assignment of a classification of **x **to class C1 or class C2.

Although SVM can be extended effectively to non-linear cases using nonlinear feature maps *ϕ *and resulting kernel matrices, we only consider linear version of SVM in this study (so that *ϕ*(**x**) = **x**).

Thus we used the linear kernel of SVM in the Spider package, with trade-off parameter *C *= 1 for all analyses

#### K nearest neighbors (KNN)

KNN is a simple and fundamental nonparametric method for classification [29], often a first choice when there is little prior knowledge about the data. Our KNN classifier is based on the Euclidean distance between a test point **x **to be classified, and a set of training samples ${\left\{x_{i}\right\}}_{i=1}^{n}$ with known classification. The predicted class of the test sample is assigned as the most frequent true class among the *k *nearest training samples. As a result, performance is more sensitive to noise in high dimensional data, which can greatly influence the relative positions of sample points in space.

We used a linear kernel with KNN (which maintains the linear geometry of the feature space *F*) in the Spider package in combination with various feature selection algorithms. For this study the number of nearest neighbors is set to *k *= 3.

#### K-TSP

The TSP classifier uses the one gene pair that achieves the highest Δ_ij _score (see above), and makes a prediction based on a simple rule for classes C1 and C2: given *P*(*R*_*i *_<*R*_*j *_| *C*1) >*P*(*R*_*i *_<*R*_*j *_| *C*2), for a new sample x, if *R*_*i*, new _<*R*_*j*, new _choose C1; and otherwise C2.

To make the classifier more stable and robust, Tan, et al. introduced the *k-*TSP algorithm [17], which builds a classifier using the *k *disjoint top-scoring pairs that yield the best Δ*_ij _*scores. Each pair votes according to the rule above, and the prediction is made according to an unweighted majority voting procedure (hence *k *must be an odd number). As for the parameter *k*, it is determined by cross-validation as described by Tan [17]. Briefly, in the case of LOOCV where there is only a training set available, a double loop is used, with an outer loop for estimating the generalization error, and an inner loop for estimating *k*. When there is an independent test set, however, only a single loop is used, and *k *is determined by the size of the subset of pairs that achieves the lowest error rate in the training set. We use the Perl version of *k-*TSP for comparison of its performance with other classifiers.

### Evaluation of classification performance

To avoid the introduction of any bias, the training of the classifier as well as the choice of the number of features (genes) and selection of features is strictly done in the training set, using either a dedicated training set when there is an independent test set available, or a number of training subsets separate from test sets in the case of 5-fold cross validation. During the training phase, standard leave-one-out cross validation (LOOCV) is used. Specifically, each of the *n *samples is predicted by the classifier trained on the remaining *n*-1 observations and the classification error rate is estimated as the fraction of the samples that are incorrectly classified. Thus as the first step in the training stage, we classify each left out sample at progressive levels of the ordered gene list (e.g. first 5, first 10, etc.), generated by a feature ranking algorithm from the remaining *n*-1 samples (note that for each iteration the selection level, i.e., number of genes, is fixed, though the features themselves vary as the left out sample changes). We then compute the LOOCV estimate at each gene selection level, and select the one achieving the minimum LOOCV error rate as defining the optimal size of a gene subset. Subsequently, the performance of the classifier, built on the entire training set using the optimized gene selection level, is evaluated on a separate test set (either a dedicated set or a left-out data subset) whose information has not been used in training the classifier, yielding a test error rate.

In simulations, we generated independent training and test sets in each experiment, and the performance estimate was averaged over the test error rates of all of the experiments. In real datasets, we made use of an independent test set when it was available from the original data, using a single test error rate as the estimate of performance; otherwise we performed 5-fold cross-validation and averaged the results of test error rates from two 5-fold experiments.

In all cases we used LOOCV for the training part, done so that the one left-out sample was not included in the feature selection procedure. Another often-used choice would have been 10-fold cross-validation, as suggested by a number of studies [19,23], due to less computational cost and possibly lower variance than LOOCV.

### Availability and requirements

Project name: *k*-TSP+SVM

Project home page: http://math.bu.edu/people/sray/software/prediction/

Operating system(s): Window XP, Window 7

Programming language: Matlab

Other requirements: Spider MachineLearning Package (provided)

License: free for academic use

## Authors' contributions

PS initiated the project, designed the study, carried out the analyses and drafted the manuscript. MK and SR contributed to the experimental design. SR developed the simulation code. QZ wrote the Matlab code and implemented the integrated scheme with PS. MK contributed to the interpretation of results and participated in drafting the manuscript. All authors read and approved the final manuscript.

## Supplementary Material

Additional file 1 **Table for Figure 4**. A table containing the simulation results for Figure 4. Click here for file
