# Supplementary material for: Top scoring pairs for feature selection in machine learning and applications to cancer outcome prediction
Source: BMC Bioinformatics. 2011 Sep 23;12:375. doi: 10.1186/1471-2105-12-375 (PMC3223741; doi:10.1186/1471-2105-12-375)
Supplement: Additional file 1 — Table for Figure 4. A table containing the simulation results for Figure 4. [file 1471-2105-12-375-S1.DOC]

# Table for figure 4

The classification error rate (mean ± SE) on Data-I ($\rho$= 0.45), with the training sets containing various sample sizes (n=25, 50, 75, 100). The results are averaged from 50 simulation experiments.

| Dataset | **Data structure** | | | | **Classification error rate on the test set (%)** | | | | | |
| --- | --- | --- | --- | --- | --- | --- | --- | --- | --- | --- |
| Training size | Signal genes | Variance | Correlation ρ | Signal vector | TSP | *k*-TSP | SVM | *k*-TSP + SVM | Fisher + SVM | RFE + SVM |
| N=25 | 10% | Fixed unit | 0.45 | $\mu_{3}$ | 45.7 ± 1.5 | 41.2 ± 1.0 | 40.1 ± 1.2 | 43.6 ± 1.1 | 46.9 ± 1.0 | 43.9 ± 1.1 |
| N=50 | 10% | Fixed unit | 0.45 | $\mu_{3}$ | 38.7 ± 1.3 | 33.2 ± 1.1 | 32.3 ± 0.9 | 33.6 ± 1.2 | 40.8 ± 1.4 | 32.5 ± 1.2 |
| N=75 | 10% | Fixed unit | 0.45 | $\mu_{3}$ | 35.3 ± 1.2 | 23.7 ± 0.8 | 25.4 ± 0.9 | 22.9 ± 1.0 | 30.8 ± 1.4 | 26.1 ± 1.0 |
| N=100 | 10% | Fixed unit | 0.45 | $\mu_{3}$ | 34.0 ± 1.0 | 21.7 ± 0.8 | 21.4 ± 0.9 | 15.8 ± 0.9 | 21.8 ± 1.0 | 21.0 ± 1.0 |
